# Emergency department use and barriers to wellness: a survey of emergency department frequent users

**DOI:** 10.1186/s12873-017-0126-5

**Published:** 2017-05-10

**Authors:** Lauren E. Birmingham, Thaddeus Cochran, Jennifer A. Frey, Kirk A. Stiffler, Scott T. Wilber

**Affiliations:** 1 Department of Emergency Medicine, Summa Health System, 525 E. Market Street, Akron, OH 44304 USA; 2 Department of Health Policy and Management, College of Public Health, Kent State University, 750 Hilltop Drive, Kent, OH 44240 USA; 3 Department of Emergency Medicine, Northeast Ohio Medical University, 4209 Route 44, Rootstown, OH 44272 USA

**Keywords:** Emergency department, Frequent user, Survey

## Abstract

**Background:**

There is no common understanding of how needs of emergency department (ED) frequent users differ from other patients. This study sought to examine how to best serve this population. Examinations of why ED frequent users present to the ED, what barriers to care exist, and what service offerings may help these patients achieve an optimal level of health were conducted.

**Methods:**

We performed a prospective study of frequent ED users in an adult only, level 1 trauma center with approximately 90,000 visits per year. Frequent ED users were defined as those who make four or more ED visits in a 12 month period. Participants were administered a piloted structured interview by a trained researcher querying demographics, ED usage, perceived barriers to care, and potential aids to maintaining health.

**Results:**

Of 1,523 screened patients, 297 were identified as frequent ED users. One hundred frequent ED users were enrolled. The mean age was 48 years (95% CI 45–51). The majority of subjects were female (64%, 64/100, 95% CI 55–73%), white (61%, 60/98, 95% CI 52–71%) and insured by Medicaid (55%, 47/86, 95% CI 44–65%) or Medicare (23%, 20/86, 95% CI 14–32%). Subjects had a median of 6 ED visits, and 2 inpatient admissions in the past 12 months at this hospital. Most frequent ED users (61%, 59/96, 95% CI 52–71%) stated the primary reason for their visit was that they felt that their health problem could only be treated in an ED. Transportation presented as a major barrier to few patients (7%, 7/95, 95% CI 3–14%). Subjects stated that “after-hours options, besides the ED for minor health issues” (63%, 60/95, 95% CI 53–73%) and having “a nurse to work with you one-on-one to help manage health care needs” (53%, 50/95, 95% CI 43–63%) would be most helpful in achieving optimal health.

**Conclusion:**

This study characterized ED frequent users and identified several opportunities to better serve this population. By understanding barriers to care from the patient perspective, health systems can potentially address unmet needs that prevent wellness in this population.

**Electronic supplementary material:**

The online version of this article (doi:10.1186/s12873-017-0126-5) contains supplementary material, which is available to authorized users.

## Background

Frequent ED users are a common topic in emergency medicine and health policy literature. Frequent ED users make up a large proportion of ED visits [1, 2], use a large number of medical resources [3, 4], and generally carry a high disease burden [5]. Previous studies have used various cut-offs for the number of annual visits, however 4 or more visits to the ED has been established as an effective value to identify frequent users [6]. Frequent ED users make numerous visits to their primary care physician [4, 5], though some studies have found that these patients change primary physicians more often than their non-frequent ED using peers [5, 6]. This patient population has higher rates of mental health and substance abuse disorders [1, 4, 6]. Fuda and Immekus determined that while frequent users make up only 1% of the patient population, they account for 17.6% of ED visits [1]. Given the high utilization rates and associated high costs, ED frequent users have historically been targeted in efforts to reduce ED crowding and costs [7].

While there is an extensive body of literature describing characteristics of ED frequent users, there is limited input from the patients regarding what services they may require to achieve the level of health they want. Our study characterizes frequent ED users, including their reason for presenting to the ED and identifies perceived barriers to care from the users’ perspective. Additionally, these patients were asked what additional service offerings, if provided by the hospital or ED would help them achieve a more optimal level of health. There is not currently a common understanding of the unique needs of ED frequent users that lead to their high ED utilization. This study helps to explain these needs.

## Methods

### Study design

We performed an Institutional Review Board approved prospective study of frequent ED users. Participants were administered a piloted structured interview by a trained researcher querying demographics, ED usage, presence of chronic disease, perceived barriers to care, and potential aids to maintaining or improving health. The survey is provided in Additional file 1: Appendix I. Other specific areas the survey discussed were what services the hospital could provide that patients thought would help them achieve an optimal level of health, self-rated health status, as well as the reason for the ED visit on the day of the survey. The research team developed and revised the survey—piloting the survey on patients prior to the start of the survey to ensure questions were easily and correctly interpreted by the intended population. Visual aids were used for multiple choice questions to assist patients. Patients were enrolled when study staff was available. To ensure a representative sample was drawn, researchers enrolled patients at a variety of day and time intervals, including coverage for all hours of the day and days of the week during a 6-month period. A screening log was maintained to ensure patients were not surveyed more than once.

The electronic registration system was used to identify current ED patients who are ED frequent users defined as those who had presented to the ED four or more times in the past 12-months, not including the day of the screening. All screened patients were recorded in a log which contained demographic and utilization data. The screening log data was used to calculate differences between frequent and non-frequent ED users in demographics and utilization rates, as reported in Table 1.

**Table 1:** Non-frequent versus all-frequent Emergency Department users

	All		Non-frequent user		All frequent user		
	*n* = 1523		*n* = 1226		*n* = 297		
	Mean	Std Dev	Mean	Std Dev	Mean	Std Dev	*p*-value
Age	52.62	19.84	53.33	20.28	49.84	17.69	0.0035
	Median	IQR (Range)	Median	IQR (Range)	Median	IQR (Range)	
# Inpatient Visits	0	0-1 (0–16)	0	0-0 (0–8)	1	0-3 (0–16)	<0.0001
# Observation Visits	0	0-0 (0–9)	0	0-0 (0–3)	0	0-1 (0–9)	<0.0001
# Emergency Visits	1	0-3 (0–45)	0	0-1 (0–3)	6	5-9 (4–45)	<0.0001
	*n*	%	*n*	*%*	*n*	*%*	
Sex (n, %)							0.0176
Female	874	57.69%	684	56.25%	189	63.85%	
Male	641	42.31%	532	43.75%	107	36.15%	
Race (n, %)							0.0335
Black	438	29.92%	332	28.28%	106	36.55%	
White	966	65.98%	791	67.38%	175	60.34%	
Asian	29	1.98%	26	2.21%	3	1.03%	
Other	31	2.12%	25	2.13%	6	2.07%	
Insurance Status (n, %)							<0.0001
Insured (any)	1183	90.86%	924	89.28%	259	97.00%	
Not insured (self-pay or no insurance)	119	9.14%	111	10.72%	8	3.00%	
Payer (n, %)							<0.0001
Medicare	355	27.22%	296	28.60%	59	22.10%	
Medicaid	428	32.80%	279	26.96%	149	55.81%	
Dual-eligible	98	7.53%	69	6.67%	29	10.86%	
None or Self-pay	119	9.14%	111	10.72%	8	3.00%	
Private	302	23.20%	280	27.05%	22	8.24%	

Exclusion criteria included: (1) previous participation in the study, (2) being less than 18 years of age, (3) presentation to the ED for an acute psychological emergency (e.g., suicide attempt), and (4) not speaking English, or being otherwise unable to speak (e.g., intubation). Once eligible frequent users were identified, the attending physician was approached for permission to interview the patient. If the attending physician agreed to have their patient interviewed, the researcher asked the patient if they would like to participate in a study about his/her healthcare use and how the hospital could improve care delivery. The researcher administered the questions orally and recorded responses prior to patient discharge from the ED.

### Study setting and population

The study was conducted in an adult-only, urban, level 1 trauma center with an annual census of approximately 90,000 visits per year. The department is affiliated with a medical university and has a 3 year emergency medicine residency program. The department in staffed by board certified/board eligible emergency physicians, residents, nurse practitioners, and physician assistants. Patients were enrolled from February to July 2015.

### Data analysis

Results were tabulated and analyzed in Microsoft Excel and SAS 9.3 (SAS Institute, Cary, NC). T-tests, chi-square, and Wilcoxon-Mann–Whitney tests were conducted at the 0.05 level of significance to determine if significant differences existed between the frequent user and non-frequent user populations. Given that this was an exploratory analysis, no adjustment was made for multiple comparisons. The sample size was calculated to accommodate a 95% confidence level and a +/− 10% confidence interval. Percentages and counts are reported for the interview data and demographic information is reported from the screening log.

## Results

**Fig. 1:**
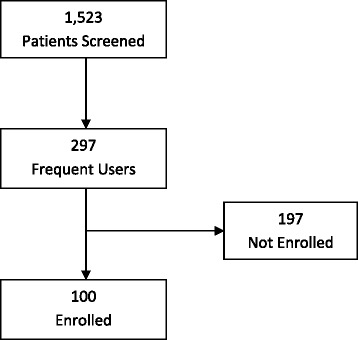
Patient flow chart

Of 1,523 screened ED patients, 19.5% (297/1523) were identified as ED frequent users. One-hundred ninety seven (197) were excluded or refused to participate. One hundred frequent ED users were enrolled (Fig. 1).

Table 1 summarizes descriptive statistics for the entire screened population and compares the entire ED frequent user population to the non-frequent user population. Frequent ED users were placed in observation or admitted more often than non-frequent users (*p* < 0.0001). ED frequent users were more frequently female (*p* = 0.0176), black (*p* = 0.0335), and insured (*p* < 0.0001) than non-frequent ED users. ED frequent users had a greater proportion of individuals with Medicaid and dual-eligible payers.

Enrolled frequent ED users were generally not significantly different from non-enrolled frequent ED users. However, the groups did differ on the number of observation visits, as presented in Table 2.

**Table 2:** Enrolled vs. Non-enrolled emergency department frequent users

	Enrolled Frequent User		Non-enrolled Frequent Users		*p*-value
	*n* = 100		*n* = 197		
	Mean	Standard Deviation	Mean	Standard Deviation	
Age	47.92	16.24	50.81	18.33	0.1871
	Median	IQR (range)	Median	IQR (range)	
# Inpatient Visits	1	0-3 (0–10)	1	0-3 (0–16)	0.9001
# Observation Visits	1	0-2 (0–7)	0	0-1 (0–9)	0.0207
# Emergency Visits	6	4-8.5 (4–37)	6	5-9 (4–45)	0.8123
	*n*	*%*	*n*	*%*	
Sex (n, %)					0.9697
Female	64	64.00%	125	63.78%	
Male	36	36.00%	71	36.22%	
Race (n, %)					0.8291
Black	36	36.73%	70	36.46%	
White	60	61.22%	115	59.90%	
Asian	0	0.00%	3	1.56%	
Other	2	2.04%	4	2.08%	
Insurance Status (n, %)					0.7151
Insured (any)	83	96.51%	176	97.24%	
Not insured (self-pay or no insurance)	3	3.49%	5	2.76%	
Payer (n, %)					0.9378
Medicare	20	23.26%	39	21.55%	
Medicaid	47	54.65%	102	56.35%	
Dual-eligible	8	9.30%	21	11.60%	
None or Self-pay	3	3.49%	5	2.76%	
Private	8	9.30%	14	7.73%	

Participating patients were asked to describe why they presented to ED (e.g., emergent issue, referral from primary care physician, nowhere else to go to receive treatment at this time, etc.) and what their primary reason was for coming to the ED. The responses of the 96 people who answered this question were grouped into one of ten categories. Individuals were allowed to select as many responses as they felt applied to them, and then they were asked to select the one response which best represented the primary reason they came to the ED that day. Sixty-one percent of patients stated that feeling like their health problem was emergent was the primary reason for their ED visit that day (59/96, 95% CI 51–71%), as reported in Table 3. However, 77% of ED frequent users (77/100, 95% CI 68%–85%) in this study stated that this was just one of the reasons for their visit. Referral to the ED by a patient’s regular physician represented the primary reason for ED attendance for 16% of respondents (15/96, 95% CI 9–24%). However, 28% (28/100, 95% CI 19–38%) stated that their regular doctor telling them to come to the ED was one of the reasons they came to the ED for this visit.

**Table 3:** Primary reason for emergency department visit amongst emergency department frequent users (*n* = 96)

What was the primary reason for your emergency department visit today?	*n*	*%*
My regular doctor is closed right now	1	1%
My regular doctor told me to come to the emergency department	15	16%
The emergency department costs me less money than my usual source of care to resolve my health issue	0	0%
The emergency department takes less time than my usual source of care to resolve my health issue	1	1%
The wait time in the emergency department is shorter than the wait time at my usual source of care	0	0%
I prefer not to schedule an appointment	0	0%
The emergency department is more convenient than my usual source of care	4	4%
My problem can only be addressed in a hospital/emergency department (emergent issue)	59	61%
I had nowhere else to go	16	17%

Of the self-reported chronic disease diagnoses, the most common was hypertension reported by 60% of patients (57/95, 95% CI 49–70%). Depression (58%, 55/95, 95% CI 47–68%), arthritis (45%, 43/95, 95% CI 35–56%), chronic back pain (45%, 43/95, 95% CI 35–56%), digestive problems (42%, 40/96, 95% CI 32–52%), and diabetes (36%, 34/95, 95% CI 26–46%) were other common conditions noted. Participants reported having an average of 5 chronic conditions (range 0–13).

Table 4 presents the perceived barriers to care that frequent ED users experience. A majority of frequent ED users agreed to understanding the information and directions given during medical appointments (94%, 90/96, 95% CI 87–98%) and to being able to obtain prescription medications in a timely manner (90%, 85/95, 95% CI 81–95%). Transportation presented as a major barrier to only a few patients (7%, 7/95, 95% CI 3–14%).

**Table 4:** Barriers to Care

Statement	Agree	Neither agree or disagree	Disagree
I have reliable transportation to get to my scheduled medical appointments on time	87% (83/95)	5% (5/95)	7% (7/95)
It is easy for me to make time to get to necessary medical appointments	78% (75/96)	7% (7/96)	15% (14/96)
I understand the information & directions given to me during my medical appointments	94% (90/96)	4% (4/96)	2% (1/96)
I always remember to schedule my annual check-ups, tests, and/or screenings	65% (60/92)	18% (17/92)	16% (15/92)
I can obtain my prescription medications in a timely manner	90% (85/95)	5% (5/95)	5% (5/95)
I feel like I receive better quality health care in the emergency department than I do in my usual place of care (primary care physician, free clinic, etc.)	48% (43/89)	35% (31/89)	17% (15/89)

The final part of the interview asked respondents to state whether or not certain services would be helpful to the patient, in the event the ED or health system decided to offer the service (Table 5). The most frequently cited desired service offering was an after-hours option for minor health issues besides the ED (63%, 60/95, 95% CI 53–73%). The least helpful option was online appointment scheduling, with 31% (29/95, 95% CI 21–41%) of frequent ED users stating this would be helpful.

**Table 5:** Potential Hospital Service Offerings Presented to ED Frequent Users

Service offering	No	Yes	N/A
Referral to a primary care physician	42% (40/96)	17% (16/96)	42% (40/96)
After-hours options for minor health issues besides the emergency department	35% (34/95)	63% (60/95)	1% (1/95)
A nurse to work with you one-on-one to help manage health care needs	38% (36/95)	53% (50/95)	9% (9/95)
Transportation to get to medical appointments on-time	51% (48/95)	46% (44/95)	3% (3/95)
Access to mental or behavioral health services	53% (50/95)	42% (40/95)	5% (5/95)
Online appointment scheduling	69% (66/95)	31% (29/95)	0% (0/95)
Referral to a specialist	39% (37/95)	44% (42/95)	17% (16/95)
Other	55% (52/95)	28% (27/95)	2% (2/95)

## Discussion

This study confirmed several findings from previous works. ED frequent users were found to use other medical services (e.g., inpatient and observation hospital stays) with higher frequency than non-frequent users. Additionally, ED frequent users were found to have a high burden of chronic disease—indicating that this is a medically complex population. This study expanded the knowledge base on frequent users by obtaining the opinions of ED frequent users on what health systems can do to better serve this population.

Our study confirmed previous literature findings, demonstrating that ED frequent users are typically insured, predominantly by public payers [8]. Our facility is located in a state that expanded Medicaid as part of the Affordable Care Act. As a result, it may be the case that our facility observes higher rates of Medicaid beneficiaries than facilities in states that have not expanded Medicaid eligibility. Literature has demonstrated that Medicaid expansion states have seen decreases in self-pay visits and increases in Medicaid visits, suggesting once uninsured individuals have gained insurance through Medicaid [9]. Medicaid expansion may have impacted who participated in this study. It may be the case that, as newly insured individuals learned how to use their health insurance, they may have used the ED with higher frequency than they normally would—especially if they did not have an established primary care provider.

The Centers for Disease Control and Prevention (CDC) recently published a report using nationally representative data summarizing reasons why adults present to the emergency department. In the CDC analysis of adults aged 18–64 years who use the ED, it was found that 77% reported that the seriousness of the medical problem was the reason for their most recent ED visit [10]. In our study, we found that 61% of frequent ED users stated that the primary reason for their ED visit was due to an emergent medical issue (although 77% of our frequent ED user participants reported this to be just one of the reasons they presented to the ED). The figures reported by the CDC for all adults aged 18–64 years are similar to our survey of ED frequent users—indicating that both populations potentially use the ED for perceived emergent medical problems.

Differences between our survey and the CDC report also exist. In the CDC analysis, it was reported that 12% of adults used the ED because their doctor’s office was not open. In our analysis, only 1% of the frequent ED user population reported visiting the ED because the doctor’s office was not open, but 16% reported that the doctor’s office had told them to come to the ED. In the CDC analysis, 7% of all ED users reported using the ED because they did not have access to any other provider. In our analysis, 17% of ED frequent users reported not having anywhere else to go to receive medical care as the primary reason for their visit, perhaps indicating that some members of the frequent ED user population may have gaps in access to health care providers.

While transportation was not reported to be a barrier to health by many participants, almost half of study participants said it would be helpful if some type of transportation to medical appointments was offered. This may reflect gaps in access to transportation and perhaps the need for transportation to unplanned medical visits, such as ED visits. This may be less problematic in other cities where mass transit is more developed—however, in the city where this analysis was conducted, mass transit is not very well-developed. Another major barrier to receiving medical care was being able to take time away from work and family to attend medical appointments. With most physician offices maintaining predominantly daytime hours, some ED frequent users noted that these times were not amenable to their schedules—indicating a potential need for more off-hours appointments. Furthermore, the fact that this population has a hard time making it to daytime appointments may explain some of their reliance on the ED, which is open at all times.

Frequent ED users typically have primary care physicians [2, 4, 5, 8, 11]. While the responses make it appear that 42% of frequent ED users did not think a primary care doctor would be helpful—those with already established primary care physicians often stated that being given a referral to a primary care physician would not be helpful because they already had one. Thus, these findings agree with prior publications that ED frequent users often have already have relationships with primary care providers.

Another key finding was that the most commonly desired service offering was an after-hours option for minor health issues besides the ED despite 77% of participants stating that their health problem was emergent. This may reflect a need for additional education regarding what level of care to seek, conditional upon symptoms. This could come in the form of a nurse call line, symptom cards, or other resources that could help ED frequent users make a decision as to which level of care to seek.

Some research has shown that frequent ED users have high disease burden and present to the ED with high acuity [12]—suggesting a hospital-based ED is often the proper place to receive care. Some participants may have felt other after-hours offerings would be potentially faster solutions than a busy, hospital-based ED, which may have also influenced their preference for the after-hours option for minor health problems. Whatever the reason, education or other related support services (e.g., nurse telephone line) are needed to ensure patients receive the right level of care—be it non-urgent, urgent, or emergent.

The majority of ED frequent users stated they had been diagnosed with depression, yet 53% of respondents stated that having a referral to mental or behavioral health services would not be helpful. Of the 55 people who stated they had depression, 23 of them stated that a referral to mental or behavioral health services would not be helpful. This may indicate that this need is already served in this population or a need for education on the benefits of mental and behavioral health services. Historically, mental and behavioral health services have not received the same level of insurance coverage as medical care on Medicaid and private health plans, making the cost of mental and behavioral health services a potential barrier for lower-income patients. Improvements in mental health parity policy in the Affordable Care Act may improve this discrepancy over time.

Future research will need to focus on clarifying desired service offerings. Specifically, it would be helpful to determine what factors were attractive about non-ED after-hours service offering to ED frequent users despite their current preference for ED care. Additional analyses of acuity will also determine whether after-hours services for non-emergent health issues would be a proper solution. Further inquiry into transportation needs is also needed to determine when and how this service is needed so as not to duplicate other community offerings. At this time, the distribution of how ED frequent users arrive to the ED is unknown (e.g., by taxi, ride from a friend, EMS, etc.). It is known that Medicaid offers non-emergent medical transportation (NEMT) which has proven cost-effective in some studies [13], but is used by only a small proportion of Medicaid beneficiaries [14]. The prevalence of Medicaid NEMT usage or other NEMT usage among ED frequent users is not known, thus an evaluation of NEMT as a way to better serve ED frequent users, and potentially reduce their ED reliance, is still needed.

Understanding the cost-effectiveness of interventions targeted toward improving the clinical and operational outcomes of frequent ED users will help health systems improve the health of this population with the greatest efficiency. Information on the resources that are available through insurers, hospitals, and other community partners that are currently being underutilized would be useful in guiding the design and implementation of hospital or ED based future services. This would reduce the probability of the hospital or ED duplicating already available resources in the community. This is especially important as there is a trend towards closing EDs across the nation [15]—impacting available capacity for patients who need emergent care.

### Limitations

This study had several limitations. This study was conducted at a single ED, and therefore may not be generalizable to all ED frequent users—especially to those outside of the United States where insurance and access to health care are different. This study excluded non-English speaking patients, which are a very small portion of our ED population, but who may have additional and unique barriers regarding their health care usage. Given that patients were being interviewed during their ED visit, those who were very acutely ill or unable to speak were not able to participate. Those who present to the ED with very acute illnesses may have systematically different opinions than those who present with less severe symptoms. Only 100 ED frequent users were enrolled in the study, which limits the precision of our point estimates. While we identified 297 ED frequent users, only 100 opted to participate in the study. While the unique reasons for non-participation were not collected, it may be the case that non-participating ED frequent users could have different opinions than those who participated—resulting in a potential selection bias. As Table 2 demonstrated, the two groups were similar on most measured metrics, but could potentially differ on metrics that were unmeasured. Lastly, the study questions were orally administered by research assistant and required participants to remember past events—opening the potential for both social desirability bias and recall bias. A different qualitative study methodology may alleviate some of the limitations related to social desirability bias and other limitations, and could potentially address questions about how to best serve the ED frequent user population with more precision. Lastly, given that this study was conducted in a state that expanded Medicaid under the Affordable Care Act, it is possible that the expansion created a subset of newly insured people who may have been using the ED at a higher rate than normal because they were unsure of how to navigate the health care system.

## Conclusions

Frequent ED users are more often female, black, insured through public programs, and carry a high chronic disease burden. They report using the ED because they feel they have emergent health concerns, although many would prefer after-hours alternatives to the ED. Barriers to wellness include not remembering to schedule regular preventative health check-ups and having difficulty taking time away from daily responsibilities for necessary medical care. Education and resources focused on acuity-appropriate alternatives to ED care for ED frequent users may be useful, as would greater awareness of community resources already available to this population.

This research demonstrates how hospitals can assist the ED frequent user population. The insights from ED frequent users gathered in this study can help shape programs or hospital offerings to better serve ED frequent users by taking a patient-centered approach to the solution.

## Additional file

Additional file 1: Appendix I. Survey Instrument. (DOCX 23 kb)

## Abbreviations

CDC: Centers for disease control and prevention
CI: Confidence interval
ED: Emergency department
IRB: Institutional review board
NEMT: Non-emergency medical transportation
