# Supplementary material for: Emergency department use and barriers to wellness: a survey of emergency department frequent users
Source: BMC Emerg Med. 2017 May 10;17:16. doi: 10.1186/s12873-017-0126-5 (PMC5424308; doi:10.1186/s12873-017-0126-5)
Supplement: Additional file 1: — Appendix I. Survey Instrument. (DOCX 23 kb) [file 12873_2017_126_MOESM1_ESM.docx]

**Appendix I: Survey Instrument**

Study Team Member: Study ID:

**1. Why did you decide to use the emergency department for your visit today?**

a. My regular doctor is closed right now

b. My regular doctor told me to come to the emergency department

c. The emergency department costs me less money than my usual source of care to resolve my health issue

d. The emergency department takes less time than my usual source of care to resolve my health issue

e. The wait time in the ED is shorter than the wait time at my usual source of care

f. I prefer not to schedule an appointment (I prefer to walk-in and be seen without an appointment)

g. The ED is more convenient than my usual source of care

h. My problem can only be addressed in a hospital/ emergency department (emergent issue)

i. I had nowhere else to go

j. Not Answered

**2. (If the participant answered multiple options in Q1)—Which was the primary reason for your visit today?** a b c d e f g h i

1. **During the past year, how many times have you visited the following places to receive medical care?**

| Location | Frequency in past 12 months | Not Answered |
| --- | --- | --- |
| a. An Emergency Department |  |  |
| b. Doctor’s Office |  |  |
| c. Retail Clinic (such as CVS Minute Clinic or Beuhler’s Quick Clinic) |  |  |
| d. Free Clinic (such as Open M) |  |  |
| e. Other (specify) |  |  |

**4. Have you been admitted to another hospital in the past 12-months?** Yes No Not Answered

| Facility | No | Yes | How many times? | NA | Facility | No | Yes | How many times? | NA |
| --- | --- | --- | --- | --- | --- | --- | --- | --- | --- |
| a. [redacted] |  |  |  |  | g. [redacted] |  |  |  |  |
| b. [redacted] |  |  |  |  | h. [redacted] |  |  |  |  |
| c. [redacted] |  |  |  |  | i. [redacted] |  |  |  |  |
| d. [redacted] |  |  |  |  | j. [redacted] |  |  |  |  |
| e. [redacted] |  |  |  |  | k. [redacted] |  |  |  |  |
| f. [redacted] |  |  |  |  |  |  |  |  |  |
| l. Other (specify): |  |  |  |  | m. Other (specify): |  |  |  |  |

**5. Have you visited another emergency department in the past 12-months?** Yes No Not Answered

| Facility | No | Yes | How many times? | NA | Facility | No | Yes | How many times? | NA |
| --- | --- | --- | --- | --- | --- | --- | --- | --- | --- |
| a. [redacted] |  |  |  |  | h. [redacted] |  |  |  |  |
| b. [redacted] |  |  |  |  | i. [redacted] |  |  |  |  |
| c. [redacted] |  |  |  |  | j. [redacted] |  |  |  |  |
| d. [redacted] |  |  |  |  | k. [redacted] |  |  |  |  |
| e. [redacted] |  |  |  |  | l. [redacted] |  |  |  |  |
| f. [redacted] |  |  |  |  | m. [redacted] |  |  |  |  |
| g. [redacted] |  |  |  |  |  |  |  |  |  |
| n. Other (specify): |  |  |  |  | o. Other (specify): |  |  |  |  |

| For the next questions, tell me how you much agree or disagree with the with the following statements: | Strongly agree | Agree | Neither agree nor disagree | Disagree | Strongly Disagree | NA |
| --- | --- | --- | --- | --- | --- | --- |
| 1. I have reliable transportation to get to my scheduled medical appointments on-time |  |  |  |  |  |  |
| 1. It is easy for me to make time to get to necessary medical appointments |  |  |  |  |  |  |
| 1. I understand the information and directions given to me during my medical appointments |  |  |  |  |  |  |
| 1. I always remember to schedule my annual check-ups, tests, and/or screenings |  |  |  |  |  |  |
| 1. I can obtain my prescription medications in a timely manner |  |  |  |  |  |  |
| 1. I feel like I receive better quality health care in the emergency department than I do in my usual place of care (primary care doctor, free clinic, etc.) |  |  |  |  |  |  |

| For the next questions, tell me how you rate yourself compared to other people your age: | Much better | A little better | About the same | A little worse | Much worse | NA |
| --- | --- | --- | --- | --- | --- | --- |
| 1. How would you rate your overall physical health, compared to other people your age? |  |  |  |  |  |  |
| 1. How would you rate your overall emotional or mental health, compared to other people your age? |  |  |  |  |  |  |

| 1. Have you ever been told that you have the following? | No | Yes | NA |
| --- | --- | --- | --- |
| a. Hypertension or high blood pressure |  |  |  |
| b. Heart issues: Such as: Heart attack (MI), Congestive Heart Failure (CHF), Coronary Artery Disease (CAD), Atrial Fibrillation (A-fib) |  |  |  |
| c. Stroke or TIA |  |  |  |
| d. High cholesterol |  |  |  |
| e. Arthritis |  |  |  |
| f. Chronic Back Pain |  |  |  |
| g. Connective Tissue Disease  Such as: Rheumatoid Arthritis (RA), Lupus, Ehlers-Danlos Syndrome (EDS), Epidermolysis Bullosa (EB), Marfan Syndrome |  |  |  |
| h. Osteoporosis |  |  |  |
| i. Anemia |  |  |  |
| j. Dementia or Alzheimer’s Disease |  |  |  |
| k. Depression |  |  |  |
| l. Asthma |  |  |  |
| m. COPD |  |  |  |
| n. Diabetes |  |  |  |
| o. Chronic Kidney Disease |  |  |  |
| p. Digestive Problems |  |  |  |
| q. Liver Problems |  |  |  |
| r. Peptic Ulcer Disease |  |  |  |
| s. Cancer |  |  |  |
| t. HIV/ AIDS |  |  |  |

| 1. Would the following help you achieve the level of health you want? | No | Yes | NA |
| --- | --- | --- | --- |
| a. Access to a primary care physician |  |  |  |
| b. After-hours options for minor health issues besides the emergency department |  |  |  |
| c. A nurse to work with one-on-one to help manage health care needs |  |  |  |
| d. Transportation to get to medical appointments on-time |  |  |  |
| e. Access to mental or behavioral health services |  |  |  |
| f. Online appointment scheduling |  |  |  |
| g. Referral to a specialist (such as pain management) |  |  |  |
| h. Other (write in) |  |  |  |

1. **(If participant answered multiple options in #15)—Which service do you think would help you the most?**  a b c d e f g h

**17. Do you currently have health insurance?** Yes No Not Answered

**18. If yes, what kind of health insurance do you have?**

a. Private insurer (insurance through your (or family member’s) employer, or private insurance which you have purchased such as Anthem, Medical Mutual of Ohio (MMO), Aetna, or COBRA)

b. Medicaid

c. Medicare, including Medicare advantage plans

d. Other ________________________________ (write-in)

e. Not Answered
